# Climate-Related Anxiety in Child and Adolescent Mental Health Services (CAMHS): A Survey of Clinician Perspectives in Aberdeen, Scotland

**DOI:** 10.1192/bjo.2025.10096

**Published:** 2025-06-20

**Authors:** David Cornelius, Praveen Kumar

**Affiliations:** City Hospital, Aberdeen, United Kingdom

## Abstract

**Aims:** This study aimed to assess clinician-reported prevalence of climate-related anxiety among children and adolescents in CAMHS, evaluate awareness of its impact, and explore the perceived relevance of Aberdeen’s oil and gas industry context to patient mental health.

**Methods:** A cross-sectional online survey was distributed to the CAMHS team at City Hospital Aberdeen, comprising four questions on climate-related anxiety and one open-text query. Sixteen clinicians participated. Data were collected anonymously via Microsoft Forms, with quantitative analysis of closed responses and thematic review of qualitative feedback.

**Results:** Prevalence and Impact: 37.5% (6/16) of clinicians reported encountering climate-related anxiety in patients over the past year, with 43.8% (7/16) ranking it as affecting young people “very much” or “quite so”. Conversely, 50% (8/16) deemed it “not a significant issue”.

Clinical Consideration: 93.8% (15/16) admitted they do not routinely assess climate-related concerns during patient evaluations.

Local Industry Context: Qualitative responses highlighted that Aberdeen’s status as an oil and gas hub may indirectly affect patients through familial job instability, frequent relocations, and eco-guilt (e.g., “Yes, the nature of the work means children face big changes and school moves”).

Awareness Gaps: Clinicians acknowledged systemic oversight in addressing climate-related anxiety during assessments.

**Conclusion:** Climate-related anxiety is inconsistently recognised and addressed in CAMHS practice, despite emerging cases and contextual ties to local industry stressors. Clinician responses reflect uncertainty about its significance, compounded by a lack of structured assessment protocols. These findings underscore the need for training to integrate climate-related concerns into routine evaluations, particularly in regions with economic dependencies on environmentally impactful industries. Recommendations include developing evidence-based screening tools, fostering interdisciplinary collaboration with environmental health sectors, and addressing systemic gaps to ensure holistic, context-sensitive care for young people.

